# Turning Waste into Useful Products by Photocatalysis with Nanocrystalline TiO_2_ Thin Films: Reductive Cleavage of Azo Bond in the Presence of Aqueous Formate

**DOI:** 10.3390/nano10112147

**Published:** 2020-10-28

**Authors:** Michele Mazzanti, Stefano Caramori, Marco Fogagnolo, Vito Cristino, Alessandra Molinari

**Affiliations:** Dipartimento di Scienze Chimiche e Farmaceutiche, Università di Ferrara, Via Luigi Borsari 46, 44121 Ferrara, Italy; michele.mazzanti@unife.it (M.M.); marco.fogagnolo@unife.it (M.F.); vito.cristino@unife.it (V.C.)

**Keywords:** TiO_2_, azo dye, wastewater treatment, photocatalysis, sodium formate

## Abstract

UV-photoexcitation of TiO_2_ in contact with aqueous solutions of azo dyes does not imply only its photocatalytic degradation, but the reaction fate of the dye depends on the experimental conditions. In fact, we demonstrate that the presence of sodium formate is the switch from a degradative pathway of the dye to its transformation into useful products. Laser flash photolysis experiments show that charge separation is extremely long lived in nanostructured TiO_2_ thin films, making them suitable to drive both oxidation and reduction reactions. ESR spin trapping and photoluminescence experiments demonstrate that formate anions are very efficient in intercepting holes, thereby inhibiting OH radicals formation. Under these conditions, electrons promoted in the conduction band of TiO_2_ and protons deriving from the oxidation of formate on photogenerated holes lead to the reductive cleavage of N=N bonds with formation and accumulation of reduced intermediates. Negative ion ESI–MS findings provide clear support to point out this new mechanism. This study provides a facile solution for realizing together wastewater purification and photocatalytic conversion of a waste (discharged dye) into useful products (such as sulfanilic acid used again for synthesis of new azo dyes). Moreover, the use of TiO_2_ deposited on an FTO (Fluorine Tin Oxide) glass circumvents all the difficulties related to the use of slurries. The obtained photocatalyst is easy to handle and to recover and shows an excellent stability allowing complete recyclability.

## 1. Introduction

Very large amounts of dyes are annually produced and used in different industries, including textile, leather and paper industries [1]. Approximately 50–70% of the dyes available on the market are azo compounds, some of which have been reported to be or are suspected to be human carcinogens [2,3].

In addition, considering that up to 20% of dyestuff is discharged directly into the environment, it appears immediately evident that azo dyes are hazardous pollutants of high impact.

Removal of colored pollutants is accomplished by traditional physical techniques (adsorption on activated carbon, ion exchange on resins, coagulation, etc.) [4,5,6,7]. Nevertheless, the organic dye is simply transferred from water to another phase, causing secondary pollution and requiring the regeneration of the adsorbent. On the contrary, advanced oxidation processes (AOP) are emerging, because they are able to disrupt the dye molecule through the action of generated OH radicals [8,9,10,11,12,13,14]. Among AOPs, TiO_2_-based photocatalysis has been the subject of numerous investigations since illumination of TiO_2_, dispersed in water containing a dissolved dye, usually led to almost complete decoloration of the solution [15,16,17,18,19,20]. Hydroxyl radical formation and their non-selective reaction with dyes have been supposed as the main degradation pathway. Although various analytical techniques (HPLC, GC–MS, LC–MS, ^1^H NMR, FT-IR) are available for the detection of the intermediates, the application of photocatalytic procedures for remediation of textile wastewaters remains rather limited [19]. A main drawback is that information available on reaction mechanisms involved in the degradation of dyes is still scant, and mineralization of intermediates is slower than the degradation of the parent compound. Until now, total mineralization has been observed for the photocatalytic degradation of most of the azo dyes only at long irradiation periods [21,22,23,24].

In this paper, we report that two alternative photocatalytic processes based on TiO_2_ can be operative in aqueous solutions containing an azo dye. Methyl orange (MO) and acid orange 7 (AO7) were chosen as representative azodyes. Specifically, the investigation of the fate of the photogenerated charges and the direct detection of OH radicals highlight that sodium formate is the switch between the two mechanisms. In fact, in its absence, we show several independent pieces of evidence that the known photodegradation of the dye occurs. However, the crucial presence of formate selectively transforms the azodye molecule into the corresponding anilines upon a reductive cleavage of its N=N bond. Transient measurements, ESR spin trapping, photoluminescence, C/C_0_ vs. irradiation time and ESI–MS data are in agreement with the role proposed for formate.

Although it has already been reported that illuminated TiO_2_ is able to reduce nitroaromatic molecules to the corresponding anilines [25,26,27,28,29,30], as far as we know, the one presented here is the first example concerning the possibility of reaching two simultaneous goals by photocatalysis, namely the removal of a waste from water by converting it into useful building blocks.

In addition, we circumvented all the difficulties related to the use of slurries of TiO_2_, since we deposited the semiconductor oxide onto a TCO-glass substrate [31,32], obtaining a photocatalyst that is easy to handle and to recover, which shows excellent stability, allowing complete recyclability and operation at ambient temperature and atmospheric pressure.

## 2. Experimental

### 2.1. Materials

Methyl orange (MO, Carlo Erba, Milan, Italy, >99.98%) and acid orange 7 (AO7, VWR, Milan, Italy, ≥97%) were purchased and used without further purification.

All other reagents employed in the experiments were also commercial: HCOONa (Sigma, Milan, Italy, 99.5%), spectrophotometric grade ethanol (Fluka, Milan, Italy, >99.8%), coumarin, (Sigma, >99%) and the spin trap 5,5′-dimethylpyrroline N-oxide (DMPO (Sigma, ≥97%).

### 2.2. Preparation of TiO_2_ on Glass Substrate

Conductive fluorine tin oxide (FTO) substrates (Pilkington TEC 7) were cleaned via sonication in 2-propanol for 10 min and dried under a warm air stream. A compact titania blocking underlayer was fabricated on top of the FTO by overnight hydrolysis of 0.4 M TiCl_4_ drop cast on top of the FTO slides (10 × 1 cm^2^), followed by firing in air at 450 °C for 30 min. Porous TiO_2_ films were obtained on top of the blocking underlayer by doctor blading a commercial terpineol based paste (Dyesol 18 NRT), followed by sintering at 500 °C for 45 min in air. The geometrical TiO_2_ film area, composed by anatase nanocrystals of the approximate size of 20 nm, was 1 cm^2^, and the film thickness was about 7 microns.

### 2.3. Structural Characterization

Atomic force microscopy (AFM) images were collected using a Digital Instruments Nanoscope III scanning probe microscope (Veeco-Digital Instruments, Plainview, NY, USA). The instrument was equipped with a silicon tip (RTESP-300 Bruker, Billerica, MA, USA) and operated in tapping mode. Surface topographical analysis of AFM images was carried out with a NanoScope Analysis 1.5. Scanning electron microscopy of the films was obtained with a JEOL JSM-7001F FEG-SEM (JEOL Ltd., Tokyo, Japan) scanning electron microscope (SEM) apparatus. Measurements were performed at 10.0–20.0 k eV electron beam energy, and the working distance was maintained between 3 and 10 mm. Surface morphology images were acquired in top-down and tilted modes, whereas cross-sectional analysis was performed putting the films on a 90° stub. X-ray diffraction (XRD) measurements were performed using a BRUKER D8 Advance X-ray diffractometer equipped with a Sol-X detector, working at 40 kV and 40 mA. The X-ray diffraction patterns were collected in a step-scanning mode with steps of Δ2θ = 0.02° and a counting time of 10 s/step using Cu Kα1 radiation (λ = 1.54056 Å) in the 2θ range of 3–80° using an incident grazing angle set-up. The crystal size (L) was estimated from the full width at half maximum (*FWHM*) of the intense (101) peak after correction from the instrumental broadening, by using the Scherrer Equation as follows:

(1) $$L=\frac{B\;\times \lambda }{FWHM\;\times \cos\theta }$$

where *B* = 0.9, *λ*= 1.54056 Å and *θ* is the angle at which the peak maximum is observed

### 2.4. Electrochemistry and Photoelectrochemistry

Open circuit chronopotentiometry to determine the quasi-Fermi level of TiO_2_ was performed in a three electrode cell (FTO supported TiO_2_ film/Pt/SCE (SCE = Standard Calomel Electrode) as follows: initially the TiO_2_ photoanode was positively polarized in the dark at 0.8 V vs. SCE for 300 s and then allowed to reach a nearly steady potential in the dark. This equilibration process was slow and occurred on the time scale of several hundred seconds. Usually after 400 s the dark potential is acceptably stable, and AM 1.5 G light is shone on the TiO_2_ substrate, causing generation of charge carriers (electron/hole couples), which may recombine or undergo separation and storage within the semiconductor. Owing to their strongly positive quasi-Fermi potential, holes are at least partially scavenged by the electrolyte before recombination occurs, allowing electrons to accumulate inside TiO_2_ and causing a sudden negative drift of the photovoltage. Illumination of the photoelectrode is maintained until a steady state value of the photopotential is attained, the reading of which provides the electrochemical potential of TiO_2_ vs. SCE. Upon restoration of the dark conditions, a fast (for the time scale of the experiment) decay of the photovoltage occurs owing to recombination. The same cell setup was used to record TiO_2_ cyclic voltammetries (CV) in the dark in the absence and in the presence of MO. The redox properties of the MO dye were investigated by cyclic voltammetry at a glassy carbon electrode, and potentials were referred against SCE.

### 2.5. Quantum Chemical Computation

DFT (Density Functional Theory) and TDDFT (Time Dependent Density Functional Theory) calculations were carried out with Gaussian 09A2 at the B3LYP 6–311 g,d level of theory [33]. The MO structure was pre-optimized at the PM6 level in vacuo before running the geometry optimization via DFT B3LYP 6-311 g,d in water solvent, described within the polarizable continuum model (PCM) approximation [34]. TDDFT computation was carried out on the optimized MO geometry, by considering the 10 lowest vertical singlet excitations in the presence of water (PCM).

### 2.6. Laser Spectroscopy

Transient absorption spectra were obtained under ns excitation of the third harmonic (355 nm) of a Nd/Yag Q switched laser oscillator described elsewhere [35]. The laser beam was diverged with a plano concave lens to achieve an energy density of 5 mJ/cm^2^ at the surface of a TiO_2_/FTO thin film, held at 45 degrees with respect to the laser beam. The thin film was in contact with an aqueous phase inside a spectrophotometric cell having an internal volume of ca. 3 mL. The aqueous phase consisted either of pure water or 0.1 M formate solution, in agreement with the explored photocatalytic conditions. The aqueous solutions could be purged with argon, if needed. The probe light was monochromatized (Applied Photophysics, Leatherhead, UK)) and passed through the sample; then, it was focused into the Acton triple grating monochromator (50 lines/mm) and fed to an R3896 photomultiplier biased at 450 V. A stack of two 380 nm cut off filters placed in front of the Acton monochromator prevented laser stray light from reaching the photomultiplier. ΔA vs. time traces were collected with a Teledyne LeCroy Waverunner 604Zi oscilloscope (Teledyne Technologies, Thousand Oaks, CA, USA) having an input impedance of 1 MΩ, and transferred to a PC with custom built software, which also controlled synchronization of the spectrometer elements. An acceptable S/N ratio was achieved by averaging 100 laser shots at each sampled wavelength.

### 2.7. ESR Spin Trapping

ESR spin trapping experiments were carried out with a Bruker ER200 MRD spectrometer equipped with a TE201 resonator (microwave frequency of 9.4 GHz). The samples were deaerated aqueous suspensions of TiO_2_ (Evonik) containing 5,5′-dimethylpyrroline N-oxide (DMPO, 5 × 10^−2^ M) as spin trap and sodium formate (0.1 M) when requested. The deaerated suspensions were transferred into a flat quartz cell inside a box under N_2_ atmosphere and directly irradiated (Hg medium pressure lamp with a cut off filter, λ ≥ 360 nm) in the ESR cavity. No signals were obtained in the dark or during irradiation of the solution in the absence of TiO_2_.

### 2.8. Photoluminescence Experiments

The fluorescence measurements (λ_exc_ = 332 nm and λ_emiss_ = 455 nm) were performed at room temperature with a Jobin Yvon Spex Fluoromax II spectrofluorimeter equipped with a Hamamatsu R3896 photomultiplier. Both emission and excitation slits were set at 5.0 nm during the measurements. For this kind of experiment, the FTO/TiO_2_ slide was put inside a Pyrex tube containing a deaerated aqueous solution (3 mL) of the dye (MO or AO7, C_0_ = 10 ppm), HCOONa (0.1 M, when requested) and coumarin (1 × 10^−4^ M) and then irradiated (λ ≥ 360 nm, 4 h). After irradiation, the fluorescence spectrum of 7-hydroxycoumarin, eventually formed, was recorded.

### 2.9. Prolonged Irradiations

Prolonged irradiations were carried out with a Helios Italquartz Q400 medium-pressure Hg lamp, Milan, IT. A glass cut-off filter was used (λ ≥ 360 nm). The incident flux was 2.75 × 10^16^ photons s^−1^ cm^−2^, calculated from the measured radiant power density in mW cm^−2^ [36]. UV–vis spectra were recorded with a Jasco V-630 double beam spectrophotometer. In a typical experiment, an FTO/TiO_2_ sheet was put inside a Pyrex tube of 15 mL capacity in front of the unique optical face, and a volume (3 mL) of an aqueous solution containing the dye (MO or AO7, C_0_ = 10 ppm) and, when requested, HCOONa (0.1 M) was added. The pH of this solution was 7.1. The closed test tube was firstly filled with N_2_ by bubbling the gas for 20 min, and then the FTO/TiO_2_ sheet was back irradiated for the desired period with the external Hg lamp. At the end of irradiation, the sample was transferred into a spectrophotometric cuvette, and the electronic spectrum was recorded. From the decrease in the absorption maximum of the band characteristic of this dye, the concentration of the remaining dye was evaluated, and a decay curve reporting C/C_0_ vs. irradiation time was built. Each experiment was repeated three times in order to evaluate the error. Blank experiments were run both in the dark, but in the presence of TiO_2_, and also illuminating the solution containing the dye (as described above), but in the absence of TiO_2_.

For recycle experiments, the just employed FTO/TiO_2_ sheet was thoroughly washed with water and dried in air at room temperature. Then it was used again in a subsequent experiment.

### 2.10. ESI–MS Investigation

Mass spectra were recorded using a LCQ Duo (ThermoQuest, San Jose, CA, USA), equipped with an electrospray ionization source (ESI), monitoring the precursor-to-product ion transitions of m/z 100 to 400 in negative ionization mode. A sample prepared as described in Section 2.6 was 4 h irradiated. Then the solution was analyzed. Ethanol (10% *v*/*v*) was used in place of HCOONa in order to have a nonionic hole scavenger.

## 3. Results and Discussion

### 3.1. Structural Properties

**Figure 1:**
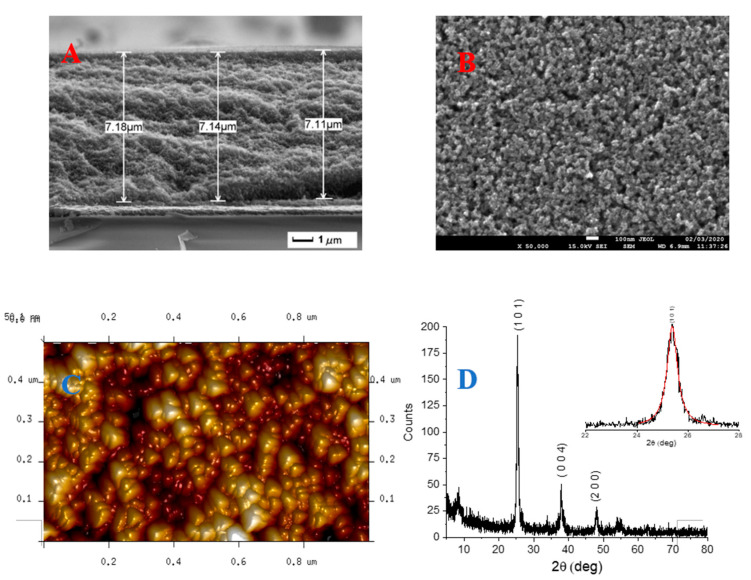
(**A**) Cross sectional SEM view of a nanocrystalline TiO_2_ thin film deposited on FTO equipped with a compact TiO_2_ blocking underlayer; (**B**) top view of the TiO_2_ surface at 50,000× magnification; (**C**) AFM imaging of the mesoporous film surface. (**D**) XRD of the thin film showing the main diffraction peak of anatase. The inset shows the 101 peak fitted with a Lorentzian function to extract the FWHM for crystal size analysis.

Figure 1A shows the cross sectional SEM imaging of a typical titania thin film used for the present work. The film displayed a porous nanocrystalline nature with a quite homogeneous thickness around 7 µm, composed of sintered particles having a size in the order of few tens of nanometers, with frequent randomly distributed larger aggregates. SEM imaging, even at 50,000× magnification (Figure 1B) did not allow each single anatase nanoparticle to be clearly resolved, which were better shown by tapping mode AFM maps (1 µm × 1 µm scanning area), where we could appreciate single particles having an approximate size of 20–30 nm together with larger aggregates resulting in lumps of 50–100 nm diameter, homogeneously distributed within the film. The sintered particles left pores and cavities which made the film permeable to the electrolyte. XRD analysis (Figure 1D) confirmed the presence of the anatase polymorph (tetragonal cell with a = b = 3.78 Å, c = 9.51 Å and 90° angles) with major reflections from (101), (004) and (200) planes. The estimation of the crystalline size (L) from the Scherrer equation provided a value of 19.8 nm, in agreement with the smallest particles observed from the scanning probe microscopies and with the nominal particle size (18 nm) contained in the commercial 18 NRT paste, confirming that each particle was a coherent scattering domain composed by single anatase crystal.

### 3.2. Energetics

**Figure 2:**
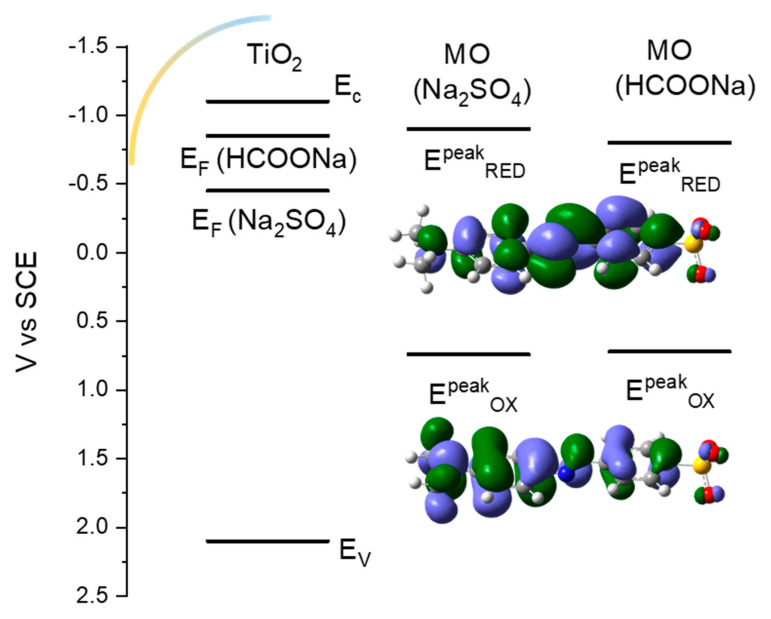
Energy levels (represented on the electrochemical scale vs. SCE) involved in the reductive degradation of the methyl orange (MO) dye at TiO_2_ photoelectrodes. The exponential Density of States (DOS) of the TiO_2_ is represented by the yellow-to-cyan curve starting at ca. −0.55 V vs. SCE, consistent with the CV results reported in the Supporting Information. In the presence of formate, the electrochemical potential of TiO_2_ matches well the first reduction peak potential of MO in the same solvent.

The energetics relevant to the photocatalytic process were explored by combining electrochemical, photoelectrochemical and quantum chemical calculations. The quasi-Fermi levels of TiO_2_ in the presence of either 0.1 M Na_2_SO_4_ or HCOONa are shown in Figure 2, together with the redox levels of MO associated to the HOMO and LUMO isodensity surfaces obtained from TDDFT calculations at the 6311 G, d level in the presence of water (PCM). The quasi-Fermi potential of TiO_2_ was obtained via open circuit chronopotentiometry under AM 1.5 G illumination (Figure S1), whereas the redox levels of the dye were obtained by cyclic voltammetry at a glassy carbon electrode (Figure S2). MO was characterized by irreversible oxidation and reduction processes, which appeared as diffusion limited waves having comparable intensity and peaking at −0.80 and +0.72 V vs. SCE in 0.1 M sodium formate supporting electrolyte. In sodium sulfate these processes maintained the same general features observed in HCOONa, with a slightly increased electrochemical gap (E^peak^_RED_ = −0.9 vs. SCE; E^peak^_OX_ = +0.74 V vs. SCE). While these processes were irreversible, meaning that chemical changes followed the charge transfer reaction, we could assume that the primary event involved the charge transfer to and from the electrode from and to HOMO and LUMO orbitals of the organic dye. We observed that the HOMO was a π orbital with a significant contribution of the donor dimethyl-amino group, whereas the lowest π* orbital with antibonding properties (LUMO) exhibited a major contribution from the diazo (–N=N–) group. The reliability of the calculation was corroborated by the fair match of the computed vertical transitions with the experimental spectrum in water (Figure S3). The contribution of the diazo group to the LUMO suggests that electrochemical reduction of MO may indeed result in the localized cleavage of this molecular unit. MO reduction became thermodynamically feasible with illuminated TiO_2_ in the presence of sodium formate. Under such conditions, efficient hole scavenging by HCOO^–^ allowed electron build up within the semiconductor, resulting in a large negative drift (absolute amplitude ca. 1.2 V) of its electrochemical potential (E_F_) up to −0.85 V vs. SCE, matching well the reduction peak of MO in the same electrolyte (Figure S4). CVs reported in Figure S5 indicated that under steady illumination, most of the trap states (oxygen vacancies) below the conduction band edge, evidenced by a pre-wave starting at ca. −0.5 V vs. SCE and peaking at ca. −0.7/−0.8 V vs. SCE (Figure S5), may have become occupied and acted as an electron reservoir for the intended reduction process. By contrast, in sodium sulfate, hole scavenging by water remained in competition with charge recombination. Hence the TiO_2_ quasi-Fermi level under AM 1.5 G illumination only reached −0.45 V vs. SCE, barely at the onset of the reduction processes of Figure S6, leading to an insufficient energy overlap between the TiO_2_ reducing states and the electron acceptor levels to drive an efficient reductive photocatalysis.

The direct clear observation of the dark electrocatalytic reaction between the TiO_2_ electrode and MO in solution was, however, quite elusive, owing to a combination of factors which include (i) the large (super)capacitive response of the porous electrode; (ii) the low solubility of MO in water (10^−3^M); and (iii) slow (for the time scale of cyclic voltammetry) electron transfer kinetics. Only at 10 mV/s did the cyclic voltammetry of the TiO_2_ film in the presence of 10^−3^ M MO show a weak intermediate wave, which could be assigned to MO reduction at the TiO_2_ surface (Figure S5).

### 3.3. Transient Spectra of TiO_2_ Thin Films

**Figure 3:**
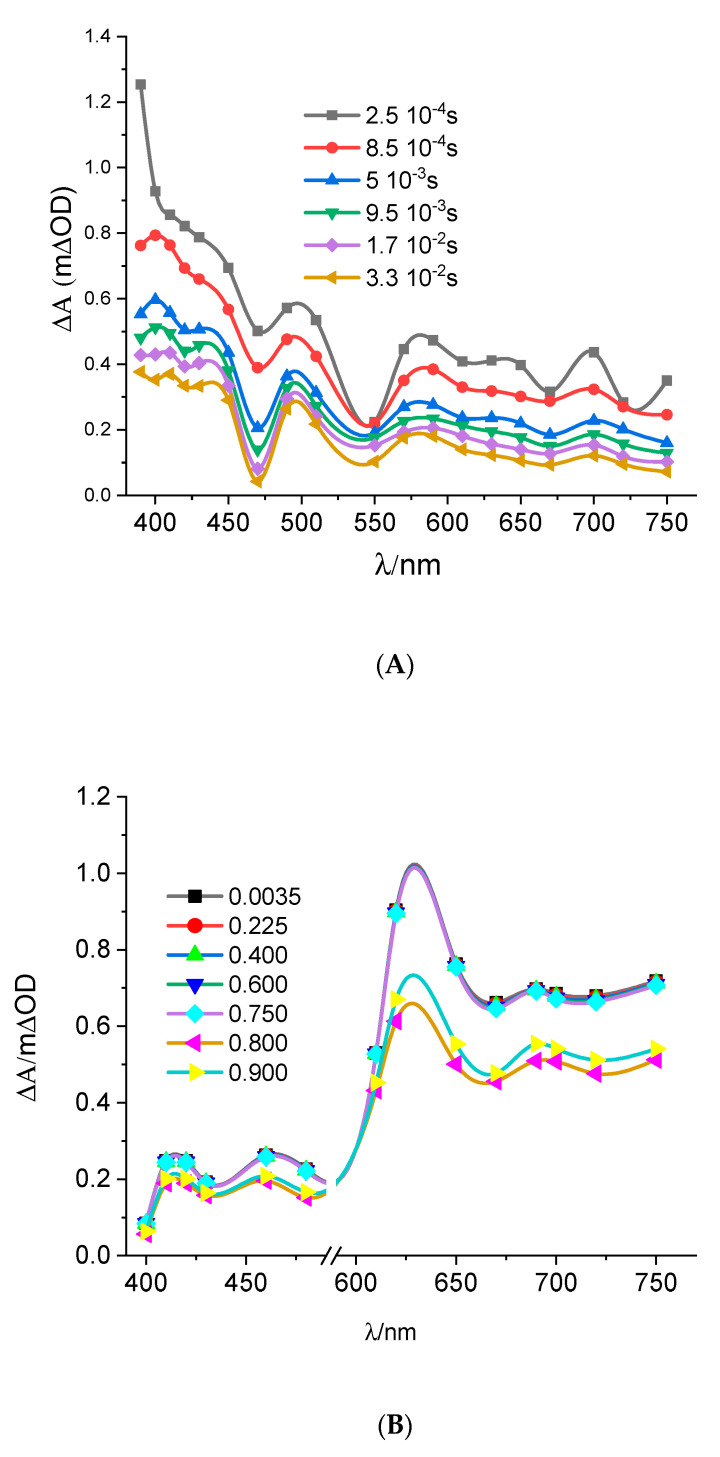
Transient spectra of TiO_2_ thin films in contact with aqueous solutions under aerated conditions. Laser excitation wavelength 355 nm, 5 mJ/cm^2^/pulse. (**A**) Pure water: difference spectra were sampled at delays from 0.25 ms to 0.33 s. (**B**) Aqueous solution of formate (0.1 M): difference spectra were sampled at delays from 3.5 ms to 0.9 s.

After having obtained an insight on the interfacial energetics involved in the photocatalytic reduction of MO, we explored the recombination kinetics with optical transient spectroscopy. Transient spectra of TiO_2_ thin films, collected in the 0.1 ms–s timeframe, were related to the simultaneous presence of trapped charge carriers having opposite sign (electrons and holes). In the presence of pure water, the spectra showed a stronger absorption in the blue visible region, which decreased by moving to the red (Figure 3A). The overall spectral shape was maintained throughout the whole timeframe, spanning two orders of magnitude, from 0.25 ms to 0.033 s, during which a general decrease of the amplitude of the transient signals was observed, according to a multiexponential decay (Figure S7). The half-life (t_1/2_) of the decays in both the blue and red parts of the visible region were comparable, about 0.033 s (0.036 s at 420 nm, 0.032 s at 720 nm). According to Bahnemann et al. [37] we assigned the blue absorption to trapped holes which could result in the formation of surface bound OH radicals. Pulse radiolytic experiments have confirmed that the ^•^OH radical absorption rises in the UV region and extends in the visible up to 470 nm [38], while earlier experiments by laser flash photolysis [39] pointed out the 420 nm absorption that we also observed as a shoulder in the spectrum reported in Figure 3A.

The broad long wavelength features were instead assigned to absorption of trapped electrons, the half-life of which nearly doubled in the absence of oxygen, which acted as an electron scavenger, affording a t_1/2_ in the order of 0.07 s, as evidenced from the 720 nm kinetics reported in Figure S8. Upon addition of 0.1 M sodium formate, the absorption of trapped holes was reduced by a factor of ca. 90%, whereas the electron absorption was concomitantly increased and longer lived (Figure 3B). The strong absorption band between 600 and 700 nm is consistent with reports by Bahnemann et al. [37] in the presence of other holes scavengers like polyvinyl alcohol. It is interesting to observe different electron absorption features by moving to pure water to 0.1 M formate. In particular, the strong absorption centered at 630 nm was dominating in the presence of formate, while it was much less evident in pure water. Such a feature is consistent with previous research which assigns the optical transitions of trapped electrons as originating from T_2g_ → E_g_ states of Ti(III), the coordination sphere of which may reflect the presence of chelating formate anions in our case [40].

### 3.4. ESR Spin Trapping Experiments

**Figure 4:**
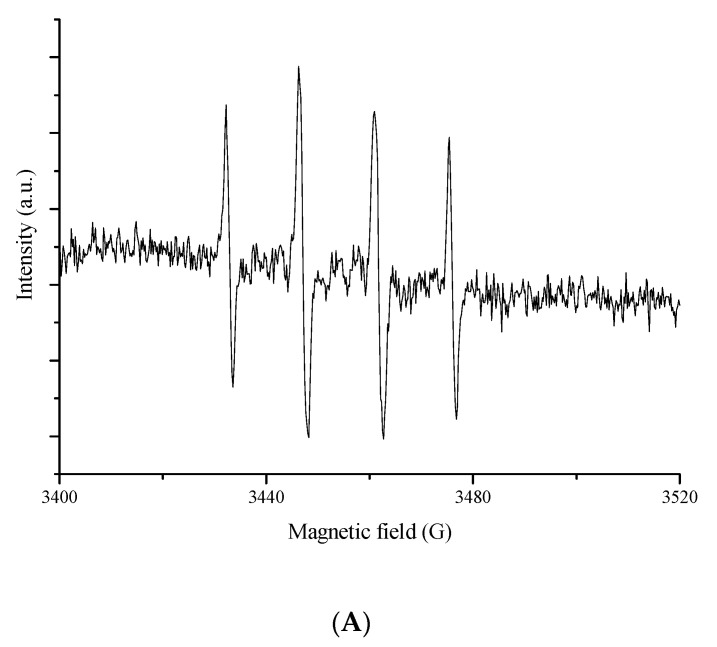

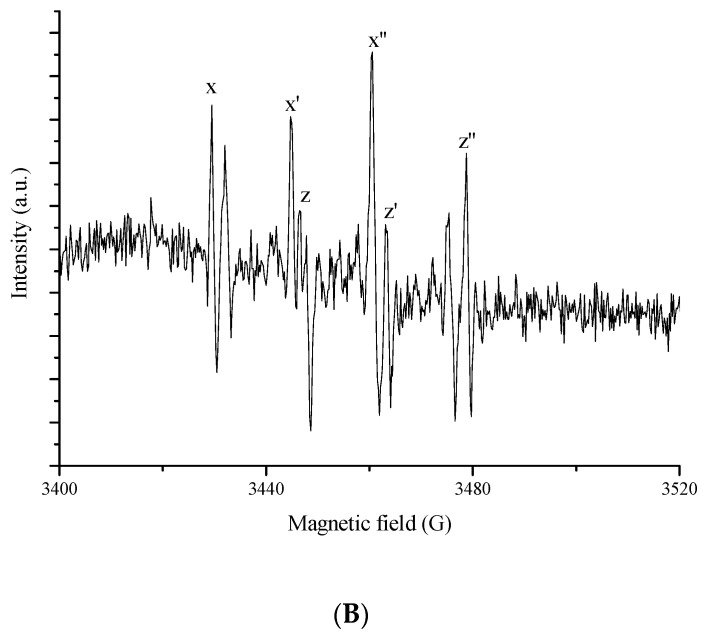
ESR spin trapping spectra obtained upon photoexcitation of TiO_2_ powder suspended in deaerated water solutions containing the spin trap DMPO (5 x10^−2^ M) (part (**A**)) and also sodium formate (0.1 M, part (**B**)).

Photoexcitation of deaerated suspensions of TiO_2_ powder in water containing the spin trap DMPO caused the formation of a quartet, 1:2:2:1 (a_N_ = a_H_ = 14.5 G), ascribable to the paramagnetic adduct [DMPO-OH]^•^ in accordance with previous investigation [41,42] (Figure 4A). Hydroxyl radicals, formed by the reaction between water on the TiO_2_ surface and positive holes, were then trapped by DMPO, according to reactions (R1) and (R2):

(R1) $$H_{2}O+h^{+}\;\to H^{+}+\;OH^{•}+e^{-}\;$$

(R2) $$DMPO+\;OH^{•}\;\to {\left[DMPO-OH\right]}^{•}$$

Interestingly, when the experiment was carried out in the presence of aqueous formate (0.1 M), the signal of the paramagnetic adduct [DMPO-OH] was no longer observed and instead a new triplet of doublets was obtained (Figure 4B). Its hyperfine splitting constants (a_N_ (x-x’) = 15.4 G, a_H_ (x-z) = 18.5 G) were consistent with the formation and trapping of a formate radical (CO_2_^–•^), as shown in reaction (R3) [43,44]:

(R3) $$HCOO^{-}+\;h^{+}\;\to \;H^{+}+\;CO_{2}^{-•}\;$$

### 3.5. Photoluminescence Experiments

The ^•^OH radical formation was investigated also by using coumarin as a fluorescent probe. In fact, the reaction between coumarin and ^•^OH radicals produced 7-hydroxycoumarin (among the possible hydroxylated products), which is a strongly luminescent compound, according to reaction (R4):
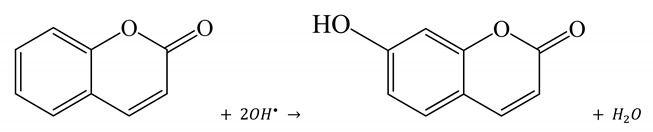
(R4)

**Figure 5:**
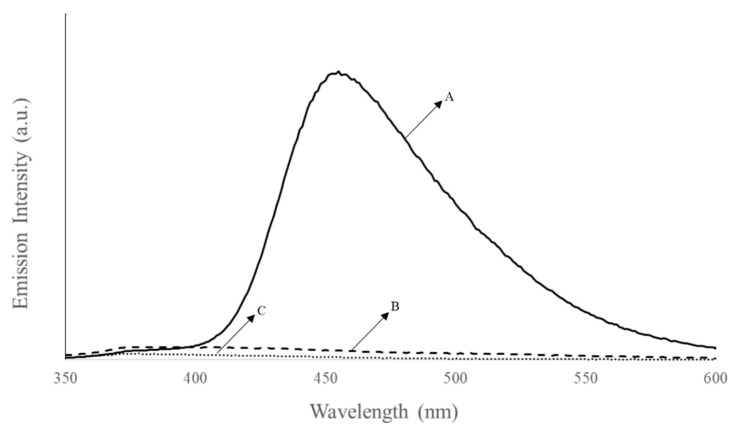
Emission spectrum of 7-hydroxycoumarin obtained upon illumination (λ > 360 nm) of FTO/TiO_2_ sheet immersed in deaerated aqueous solutions containing coumarin (1 × 10^−4^M), (A); (B) is the spectrum obtained in the same conditions as A, except that 0.1 M HCOONa was present (curve B). (C) refers to the emission of the sample before illumination.

This method has been successfully applied for the detection of hydroxyl radicals generated by photoexcited TiO_2_ [45,46]. Therefore, the FTO/TiO_2_ sheet immersed in a deaerated aqueous solution containing MO (10 ppm) and coumarin (1 × 10^−4^ M) was irradiated (λ > 360 nm, 4 h) both in the absence and in the presence of formate (0.1 M), and the emission spectrum of the irradiated solution was recorded. It was observed (Figure 5) that an important emission from 7-hydroxycoumarin was obtained in the absence of HCOONa (curve A), indicating the occurrence of reaction (R4). Conversely, in the presence of formate, fluorescence was obtained (curve B), evidencing that OH radical formation was negligible, in accordance with ESR spin trapping results. Curve B was in fact similar to the spectrum of the starting solution before irradiation, which contained unreacted coumarin (curve C).

All the previous results lead to common conclusions that are summarized in the following: transient spectra confirm that charge separation is extremely long lived in nanostructured TiO_2_ thin films, making them suitable to drive both oxidation and reduction reactions by exploiting the respective trapped charge carriers. Formate anions are very efficient in intercepting holes, inhibiting OH radical formation and allowing both the concentration and lifetime of trapped electrons to increase in order to use them for multi-electron reductive photocatalysis. On the basis of these considerations, we have experimental evidence that photoexcitation of TiO_2_ immersed in water can lead to selective reduction processes if formate is used as a hole scavenger. Moreover, protons formed as described in reaction (R3) and electrons promoted in the conduction band could perform hydrogenation reactions. In the following, we explore this possibility considering reductive cleavage of N=N bonds in MO and in AO7, chosen as representative azo dyes.

### 3.6. Prolonged Irradiation Experiments

**Figure 6:**
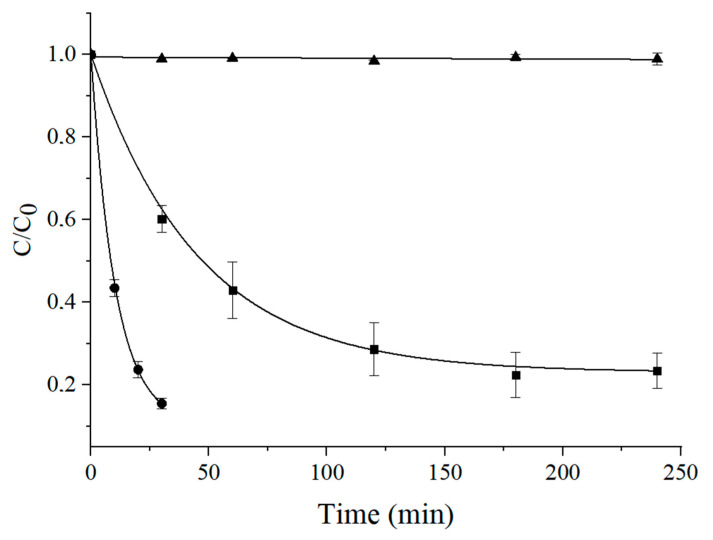
C/C_0_ ratio vs. time profiles obtained upon irradiation (λ ≥ 360 nm) of an FTO/TiO_2_ slide (active TiO_2_ geometrical area =1 cm^2^) immersed in deaerated aqueous solutions containing HCOONa (0.1 M) and MO (full squares) or AO7 (full circles). C_0_ = 10 ppm. Full triangles: the same solutions irradiated in the absence of FTO/TiO_2_.

An FTO/TiO_2_ slide, having a TiO_2_ coated area of 1 cm^2^, immersed in a deaerated aqueous solution containing MO (C_0_ = 10 ppm) and HCOONa (0.1 M), was irradiated from the FTO side (λ ≥ 360 nm). This illumination geometry is referred to as back illumination mode. During irradiation, a loss of the solution color that corresponded to a decrease of the absorption band of MO (maximum at 464 nm), having a charge transfer (CT) character, was observed. Figure 6 reports a C/C_0_ ratio of MO as a function of irradiation time. It was observed that after 240 min irradiation, about 80% of the starting dye had disappeared from the solution (full squares). Control experiments pointed out that this result was exclusively ascribable to photocatalytic activity of TiO_2_, since irradiation of the dye solution in the absence of semiconductor led to no spectral variations (i.e., no photolysis, Figure 6 full triangles). Moreover, no spectral variation was observed when the nanocrystalline TiO_2_ thin film was put in contact with the dye solution and kept in the dark.

An analogous behavior was observed when AO7 was used instead of MO. C/C_0_ vs. time reported in Figure 6 (full circles) showed that around 90% of this dye disappeared from the solution after only 30 min irradiation.

Curves similar to those of Figure 6 were also obtained in the absence of formate (not shown here), where formed OH radicals were responsible for the oxidative degradation of the dye. Thus, monitoring the disappearance rate of the target dye is not the most appropriate way to establish the nature of the photocatalytic reaction. It should be taken into account that fading of the dye solution is only an indication of the interruption of conjugation.

**Figure 7:**
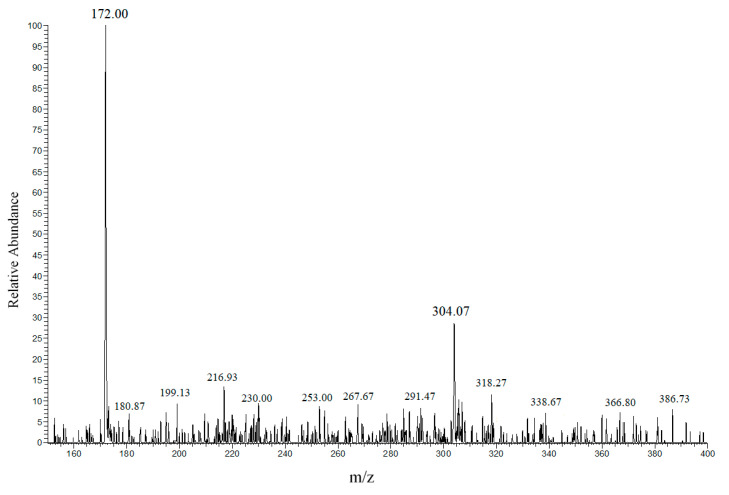
ESI–MS spectrum of the deaerated solution containing MO (10 ppm) and ethanol (10% *v*/*v*) after irradiation in the presence of FTO supported TiO_2_ (1 cm^2^). Ethanol is used here in place of formate as a non-ionic hole scavenger.

With the aim of ascertaining that the observed decrease of dye concentration is due to the reductive cleavage of the azo bond with formation of the two amine derivatives, we recorded the negative ion mode ESI–MS spectrum of the irradiated solution (Figure 7). Besides the m/z peak at 304 relative to the residual MO in its anionic form, the dominant base peak at m/z 172 corresponded to the anionic form of sulfanilic acid [47]. A similar result was obtained in the case of AO7 (Figure S9). Conversely, no evidence of the m/z 172 peak was obtained in the ESI–MS spectrum recorded after photocatalytic processing of the dye in the absence of formate (Figure S10). Detection of a peak with m/z 227 was in agreement with the formation of a byproduct whose molecular weight was consistent with hydroxyl group substitution on a phenyl ring [18]. In addition, many examples in the literature report that oxidation of aromatic organic molecules (such as drugs, pesticides) proceeds by the addition of hydroxyl radicals before ring breaking [15,21,48,49]. Thus, the pattern reported in Figure S4 is characteristic of a degradative process promoted by OH radicals.

**Scheme 1:**
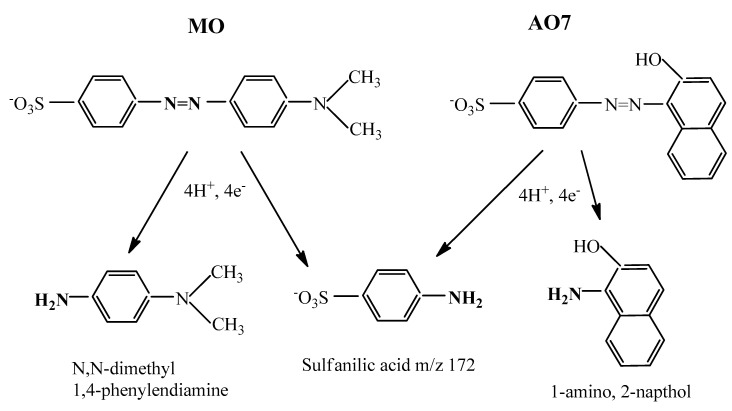
Representation of N=N reductive cleavage of MO and of AO7 with formation of amine intermediates and of sulfanilic acid.

These results give evidence that when OH production is suppressed, the two azodyes MO and AO7 undergo a hydrogenation reaction on the diazo N=N bond (via protons and electrons addition). The subsequent cleavage of this bond leads to the formation of the two corresponding amine fragments, N, N-dimethyl 1, 4-phenylendiamine (from MO), 1-amino, 2-napthol (from AO7) and sulfanilic acid (from both MO and AO7), as summarized in Scheme 1.

The photocatalytic cleavage of the diazo bond, here observed, most likely occurred at the surface of the anatase mesoporous film, with the involvement of protons provided by the oxidation of either formate or ethanol, as a result of photo-hole scavenging and of electrons stored in long living trap states inside the TiO_2_ nanoparticles, and clear evidence of electron accumulation within the TiO_2_ thin films was provided by both photoelectrochemical and spectroscopic means. Electron accumulation inside TiO_2_ was also evident to the naked eye when using TiO_2_ P25 (10 mg) nanoparticles suspended in a deaerated aqueous solution containing HCOONa (0.1 M) and irradiated with UV light (λ ≥ 360 nm). While photogenerated holes were efficiently scavenged by formate, electrons, in the absence of any acceptor, accumulated, causing the formation of Ti(III) centers, responsible for the grey–blue color of the overall suspension [50]. After this was achieved, MO was added to the blue suspension through a septum. In the following minutes, the blue color bleached and a simultaneous decrease of the absorption in the 400–500 nm region was observed in the UV–visible spectrum of the solution after centrifugation (data not shown). This result confirms the ability of MO to scavenge electrons.

The alternative proton source could be water oxidation by photogenerated TiO_2_ holes (reaction (R5)):

(R5) $$H_{2}O+\;h^{+}\;\to \;H^{+}+\;OH^{•}+\;e^{-}\;$$

However, since hydroxyl radical formation was completely inhibited in the presence of formate (see Section 3.3 and Section 3.4), we can conclude that it is formate or another organic hole scavenger, like ethanol, that provides the required protons that participate in the reductive process schematized below.

This new synthetic route occurring in water by photoexcited TiO_2_ is of particular significance; in fact, aqueous TiO_2_ photocatalysis that is usually considered an AOP useful in depollution converts here an azo dye into useful reduced intermediates, one of which, sulfanilic acid, could be directly recycled in the synthetic process of new azo dye. Moreover, HCOONa oxidation leaves no residue (it is converted to CO_2_) in the reaction environment, and the whole photocatalytic process is carried out at pH around 7 with no adjustments.

### 3.7. Recycle

**Figure 8:**
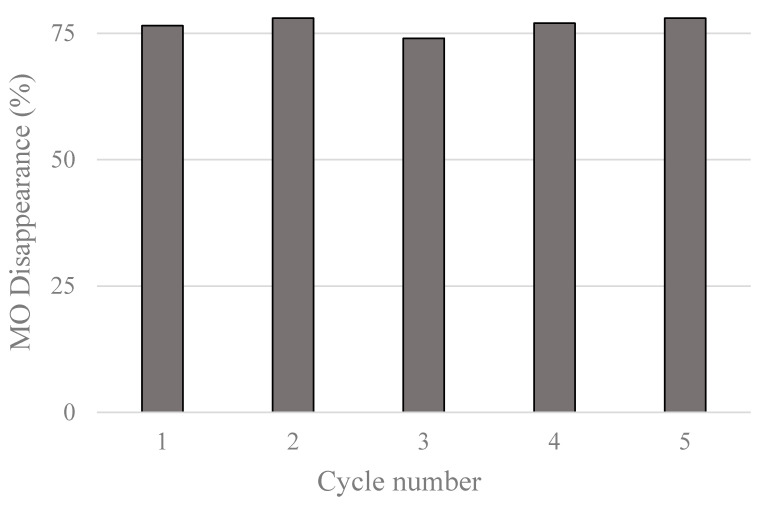
MO disappearance (%) obtained by irradiating (4 h, λ ≥ 360 nm) the same FTO supported TiO_2_ thin film during five consecutive photocatalytic runs.

Recyclability of the photocatalyst was evaluated using the same FTO/TiO_2_ slide in several consecutive photocatalytic experiments. Typically, the FTO/TiO_2_ immersed in a deaerated aqueous solution containing MO (10 ppm) and HCOONa (0.1 M) is back irradiated (λ ≥ 360 nm) for a period of 4 h. At the end of the illumination period, MO disappearance (that we established to be due to reductive cleavage of N=N bond) is spectrophotometrically evaluated. The slide is then washed with distilled water and reused in a subsequent experiment. Figure 8 reports the amount of dye (%) disappeared in five consecutive runs. It can be noted that, within the experimental error, no decrease in photocatalytic performance was observed. This result indicates that the studied photocatalytic system is perfectly stable and completely re-usable.

Moreover, three different FTO/TiO_2_ slides (all prepared with the procedure described in 2.2) were used for the same photocatalytic experiment and gave very similar results, demonstrating that also the preparation procedure provides reproducible photocatalysts.

## 4. Conclusions

This study demonstrates that the fate of azo dyes dissolved in aqueous solution in the presence of illuminated TiO_2_ can be determined by the experimental conditions. In fact, the usual photocatalytic degradation of the dye by OH radicals can be inhibited by the presence of sodium formate or of other hole scavengers that are able to release protons upon oxidation.

Laser flash photolysis experiments show that charge separation is extremely long lived in nanostructured TiO_2_ thin films, making them suitable to drive both oxidation and reduction reactions. When a suitable hole scavenger, like formate, is present, holes are consumed in the sub-ms time scale, consistent with ESR spin trapping and photoluminescence experiments, demonstrating the formation of OH radicals. ESI–MS analysis of irradiated samples shows that multi-electron reduction of the azo dye on photoexcited TiO_2_ is operative. In particular, a reductive cleavage of N=N bond of the dye takes place, with formation of aromatic anilines and of sulfanilic acid, which in turn can be recovered and reused for the synthesis of new dyestuffs. Formate is also the source of required protons and its oxidation leaves no residue in the solution.

This result is of interest because wastewaters, colored by the presence of dyes, can be the starting material for the conversion of pollutants into useful products by photoexcited TiO_2_ at ambient temperature and atmospheric pressure. In addition, immobilization of TiO_2_ on FTO glasses gives a heterogeneous system that is very stable, easy to separate from the solution medium, completely recyclable for countless times and with high reproducibility in the preparation. For this, the findings presented here may provide a new highly efficient and low cost method for azo dye wastewater treatment, opening to a proper circular approach.

## Supplementary Materials

The following are available online at https://www.mdpi.com/2079-4991/10/11/2147/s1, Figure S1: Open circuit chronopotentiometry, Figure S2: Cyclic voltammetry of MO at a glassy carbon electrode, Figure S3: Experimental vs computed MO spectrum, Figure S4: Cyclic voltammetry of TiO_2_ in HCOONa, Figure S5: Cyclic voltammetry of TiO_2_ in Na_2_SO_4_, Figure S6: Cyclic voltammetry of MO at a TiO_2_ electrode, Figure S7: 430 nm kinetics in pure water, Figure S8: 720 nm decay kinetics, Figure S9: ESI-MS spectrum in AO7 (10 ppm) and ethanol (10% *v*/*v*), Figure S10: ESI-MS spectrum in MO.
