# Supplementary material for: Turning Waste into Useful Products by Photocatalysis with Nanocrystalline TiO2 Thin Films: Reductive Cleavage of Azo Bond in the Presence of Aqueous Formate
Source: Nanomaterials (Basel). 2020 Oct 28;10(11):2147. doi: 10.3390/nano10112147 (PMC7716207; doi:10.3390/nano10112147)
Supplement: Supplementary file 1 [file nanomaterials-10-02147-s001.docx]

Supporting Information

Turning Waste into Useful Products by Photocatalysis with Nanocrystalline TiO_2_ Thin Films:
Reductive Cleavage of Azo Bond in the Presence
of Aqueous Formate

Michele Mazzanti, Stefano Caramori *, Marco Fogagnolo, Vito Cristino and Alessandra Molinari *

Dipartimento di Scienze Chimiche e Farmaceutiche, Università di Ferrara, Via Luigi Borsari 46, 44121 Ferrara, Italy; michele.mazzanti@unife.it (M.M.); marco.fogagnolo@unife.it (M.F.); vito.cristino@unife.it (V.C.)

***** Correspondence: cte@unife.it (S.C.); alessandra.molinari@unife.it (A.M.)

Figure S1. XRD diffraction of TiO_2_/FTO substrate S2

Figure S2. SEM of TiO_2_ S2

Figure S3. Open circuit chronopotentiometry S3

Figure S4. Cyclic voltammetry of MO at a glassy carbon electrode S4

Figure S5. Experimental vs computed MO spectrum S4

Figure S6. Cyclic voltammetry of TiO_2_ in HCOONa S5

Figure S7. Cyclic voltammetry of TiO_2_ in Na_2_SO_4_ S5

Figure S7. Cyclic voltammetry of MO at a TiO_2_ electrode S6

Figure S9. 430 nm kinetics in pure water S6

Figure S10. 720 nm decay kinetics S7

Figure S11. ESI-MS spectrum in AO7 (10 ppm) and ethanol (10% v/v) S7

Figure S12. ESI-MS spectrum in MO S8

**Figure S1.**  XRD diffraction of the TiO_2_ thin film cast over FTO. Small-angle XRD confirmed the presence of the TiO_2_ Anatase phase (red line) and SnO_2_ (blue line) Cassiterite phase from underlying FTO.


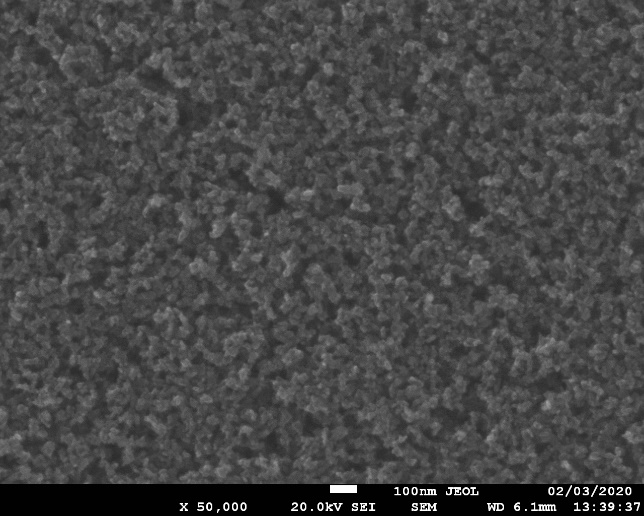

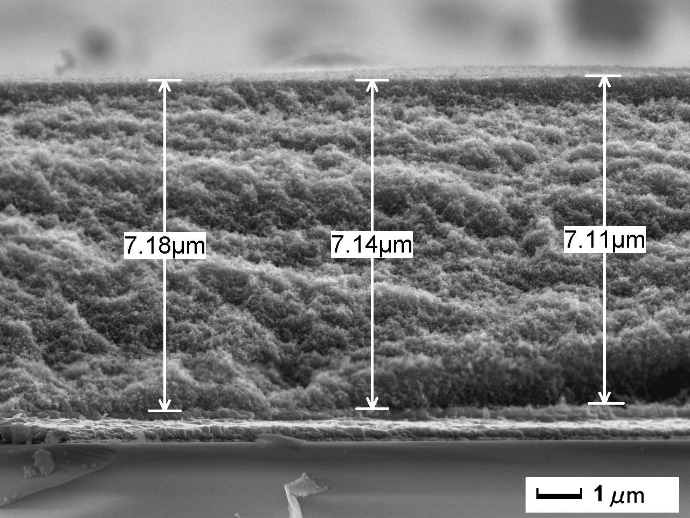


1. **(B)**

**Figure S2.** SEM imaging of a nanocrystalline TiO_2_ film. (A) cross section; (B) top view .

**Figure S3.** Open circuit chronopotentiometry of a TiO_2_ photoanode in the presence of 0.1 M Na_2_SO_4_ (red) and 0.1 M HCOONa (black) adjusted at pH 7. Note that in the presence of HCOO^-^ acting as a fast scavenger it is possible to attain a ca. twice more negative TiO_2_ photopotential, due improved accumulation of electrons inside the semiconductor.

**Figure S4.**  Cyclic voltammetry of the 10^-3^ M MO dye (20 mV/s) recorded in the presence of either 0.1 M HCOONa or 0.1 M 0.1 M Na_2_SO_4_ adjusted at pH 7. The scan direction was first in reduction (I) and then oxidation (II). The photopotential attained by TiO_2_ in the two different electrolytes is represented by the vertical dash-dot lines. Electrochemical oxidation of HCOO^-^ results in and irreversible anodic discharge at V > 1.2 V vs SCE shown by the red curve. .

**Figure S5.** Experimental vs computed MO spectrum in water (PCM). .

**Figure S6.** Cyclic voltammetry of the FTO/TiO_2_ electrode recorded under variable scan rates in the presence of 0.1 M HCOONa adjusted at pH 7. The photopotential recorded under the same electrolyte conditions is represented by the vertical dotted line. The potential shift observed with increasing scan rate is due to ohmic drop due to the non-negligible resistance of the FTO. .

**Figure S7.** Cyclic voltammetry of the FTO/TiO_2_ electrode recorded under variable scan rates in the presence of 0.1 M Na_2_SO_4_ adjusted at pH 7. The photopotential recorded under the same electrolyte conditions is represented by the vertical dotted line.

**Figure S8.** Cyclic voltammetry of an FTO supported TiO_2_ film in contact in 0.1 M HCOONa in the absence (black) and in the presence (red) of 10^-3^ M MO. The wave indicated by the arrow is consistent with the MO reduction potential in this electrolyte. The vertical bar indicates the electrochemical potential attained by the electrode under illumination, confirming the thermodynamic feasibility of the reductive process of MO at illuminated titania films.

**Figure S9.** Decay of the 420 nm Differential Absorbance observed upon 355 nm excitation of the TiO_2_ thin film in contact with plain water. The biexponential fit was performed as a guide to the eye.

**Figure S10.** Decay of the 720 nm Differential Absorbance observed upon 355 nm excitation of TiO_2_ thin film in contact with aqueous phase: aerated pure water (black); Ar purged plain water (orange); 0.1 M formate in the presence of air. .
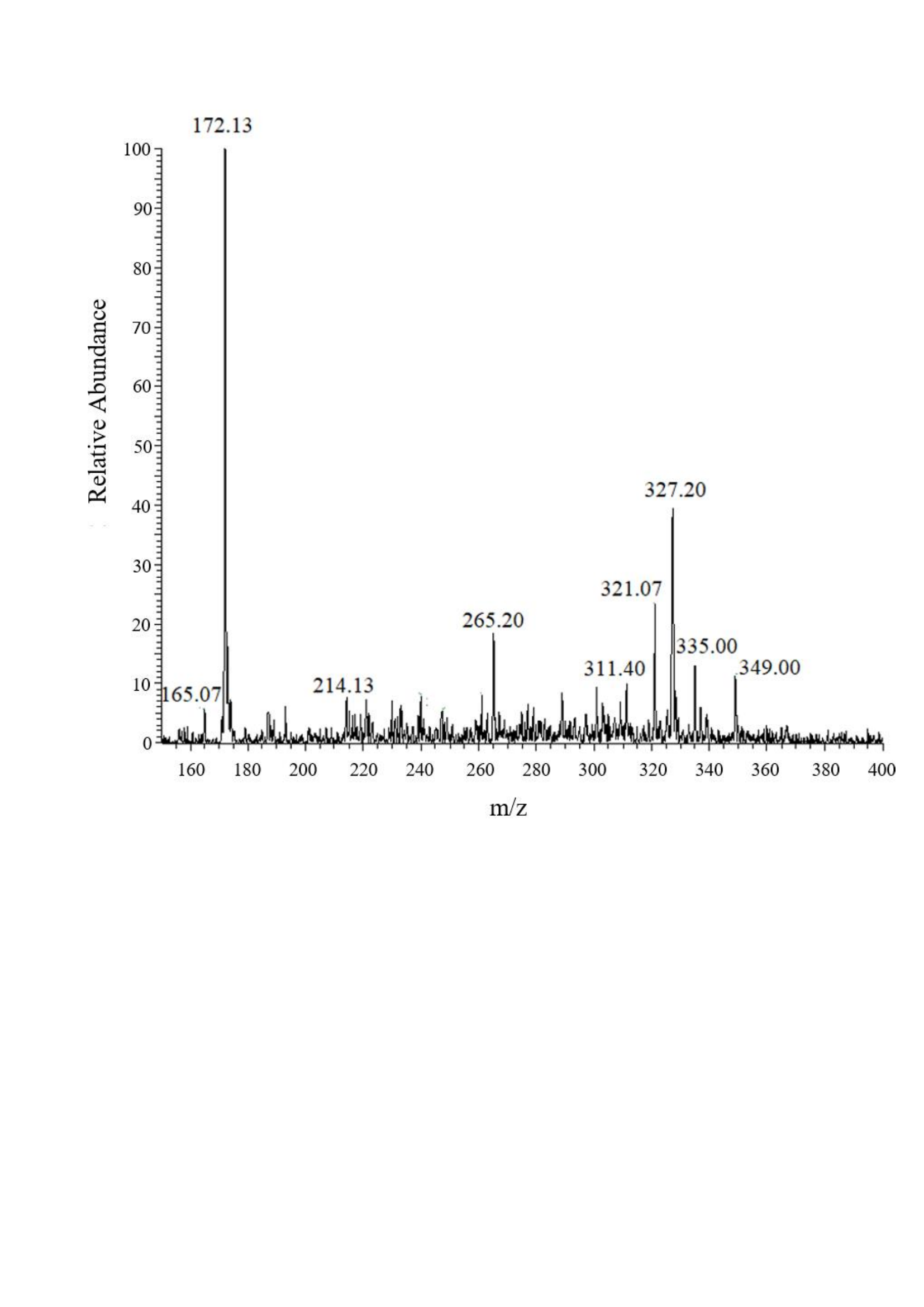


Figure S11. ESI-MS spectrum of the aqueous solution containing AO7 (10 ppm) and ethanol (10% v/v) after irradiation of FTO/TiO_2_. Ethanol is used in place of formate.


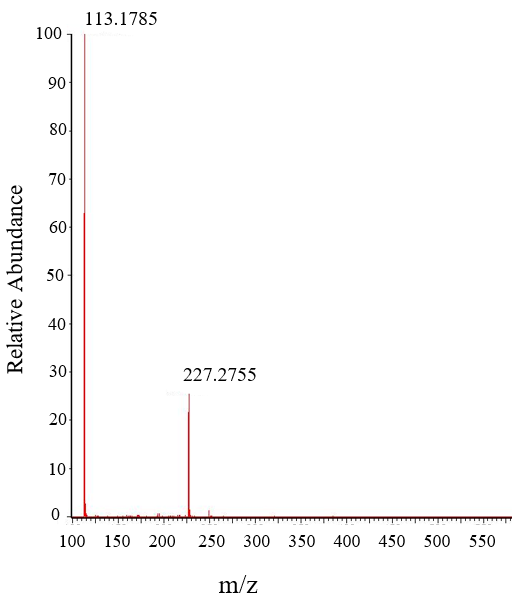


**Figure S12.** ESI-MS spectrum of the aqueous solution containing MO (10 ppm) after irradiation of FTO/TiO_2_.
